# Triiodothyronine/Free Thyroxine Ratio as a Criterion for the Differentiation Between Graves' Disease and Subacute Thyroiditis

**DOI:** 10.7759/cureus.63083

**Published:** 2024-06-25

**Authors:** Haider A Alidrisi, Nawar K Thamer, Maher A Radhi, Saad Hammadi, Farah S Abdulaziz

**Affiliations:** 1 Diabetes and Endocrinology, Faiha Specialized Diabetes, Endocrine and Metabolism Center, Basrah, IRQ; 2 Diabetes and Endocrinology, University of Basrah, College of Medicine, Basrah, IRQ; 3 Medicine, University of Basrah, College of Medicine, Basrah, IRQ; 4 Internal Medicine, University of Basrah, College of Medicine, Basrah, IRQ; 5 Radiology, Al-Mawani Teaching Hospital/University of Basrah, College of Medicine, Basrah, IRQ

**Keywords:** triiodothyronine, thyroxine (t4), hyperthyroidism, subacute thyroiditis, grave's disease

## Abstract

Background: Graves' disease (GD) and subacute thyroiditis (SAT) are important causes of thyrotoxicosis. The differentiation between these diseases is of great value because it will affect the management plan of either of them. The study aimed to assess the triiodothyronine/free thyroxine (T3/fT4) ratio as a criterion for the differentiation of hyperthyroidism due to GD and SAT.

Method: A retrospective study with database retrieval was conducted at Faiha Specialized Diabetes, Endocrine and Metabolism Center (FDEMC), Basrah, southern Iraq. Patients attending the center who presented with thyrotoxicosis due to GD and SAT from January 2010 to January 2024 were included in the analysis that was conducted from October 2023 to February 2024. For comparison between GD and SAT, the baseline thyroid-stimulating hormone (TSH), fT4 and T3 were used to calculate the fT4 ratio (fT4 level (ng/dL)/1.7 ng/dL), T3 ratio (T3 level (ng/dL)/200 ng/dL), and T3/fT4 ratio (T3 level (ng/dL)/fT4 (ng/dL)).

Results: As compared to SAT, patients with GD had a significantly lower TSH and higher T3, T3 ratio, and T3/fT4 ratio. A T3/fT4 ratio with a cutoff equal to or more than 25 had 95% sensitivity and 18.1% specificity for GD with 94.4% positive predictive value. Raising the cutoff to equal or more than 100 results in the reduction of sensitivity to 32.7% but with 100% specificity and positive predictive value.

Conclusion: The T3/fT4 ratio presents as a valuable diagnostic tool in differentiating GD from SAT, with potential applications in refining the diagnostic approach to hyperthyroidism.

## Introduction

Thyroid hormones play a role in many metabolic events such as the cardiovascular system, neurological system, immune system, bone development, energy expenditure, glucose and lipid regulation, and coagulation [1]. Hyperthyroidism is a state of hyperfunctioning thyroid gland that is characterized by various clinical manifestations related to thyroid hormone excess [2]. In a retrospective study from Basrah that included more than 17,000 patients with various thyroid diseases, hyperthyroidism constituted 6.1% [3]. The commonest cause of hyperthyroidism is Graves' disease (GD). Other causes include toxic nodular thyroid disease, trophoblastic disease, thyroid-stimulating hormone (TSH)-secreting pituitary adenoma, and resistance to thyroid hormones. These diseases are characterized by normal or increased thyroid radioactive iodine uptake (RAIU). Thyroiditis, including subacute (granulomatous and de Quervain's) thyroiditis (SAT), a disease characterized by inflammation of thyroid parenchyma and absent or low thyroid radioactive iodine uptake, can present with state thyrotoxicosis [2]. Specifying the underlying cause of thyrotoxicosis is necessary for effective management of the disease.

Guidelines for the diagnosis of GD recommend the measurement of the TSH receptor antibodies (TRAb) besides the presence of specific signs for GD such as Graves' ophthalmopathy and dermopathy [2,4]. In patients with hyperthyroidism, the clinical manifestations in the form of painful and tender thyroid gland, increased inflammatory markers (e.g., erythrocyte sedimentation rate (ESR) and C-reactive protein (CRP)), and focal or diffuse reduced colored Doppler flow on ultrasound (US) thyroid examination may help in suggesting the diagnosis of SAT [5,6].

In the absence of or unclear presence of the specific features of GD, detectable TRAb and/or increased RAIU will point to the diagnosis of GD. Measurement of TRAb is considered an excellent and cost-effective marker for GD if RAIU is not obtained. TRAb assay in our locality is done by a third-generation automated assay for TSH receptor-binding inhibitor immunoglobulin (TBII) that measures stimulating, blocking, and neutral antibodies. It is less expensive, but not specific for the thyroid-stimulating immunoglobulin that causes GD [2].

Recent observational data suggested that variation in thyroid hormones within the reference range also deteriorates outcomes in specific clinical conditions, possibly through impairment of peripheral thyroxin deiodination and downregulation of deiodinase activity [7]. The free triiodothyronine/free thyroxine (fT3/fT4) ratio has been reported as a useful indicator for differentiating SAT from GD [8]. However, these ratios are not widely used because they are derived from small sample studies. There is considerable evidence that the hyperthyroidism associated with GD is accompanied by the hyperproduction of triiodothyronine (T3) relative to thyroxine (T4) [9]. The most extreme example of this phenomenon was found in patients with the clinical feature of hyperthyroidism, a normal T4 and elevated T3, a condition that has been called "T3 thyrotoxicosis" [10].

A total T3/total T4 ratio of <20 ng/mcg in thyrotoxic patients may be considered a useful indicator of SAT [2]. Instead of total form, fT3 and fT4 measurements are more widely used because they are less affected by thyroid hormone-binding proteins and are more accessible. The fT3/fT4 ratio has been reported as a useful indicator in differentiating SAT from GD [8,11].

In our locality, the most used thyroid hormone measurements after TSH are fT4 and T3. In this study, we aimed to assess the T3/fT4 ratio as a criterion for the differentiation of thyrotoxicosis due to GD and SAT.

## Materials and methods

A retrospective study with database retrieval was conducted at Faiha Specialized Diabetes, Endocrine and Metabolism Center (FDEMC), Basrah, southern Iraq. Patients attending the center who presented with hyperthyroidism from January 2010 to January 2024 were included in the analysis that was conducted from October 2023 to February 2024.

We included adult patients (aged 18 years old or older) who presented for the first time in the center with overt thyrotoxicosis (low TSH and high fT4 and/or T3 levels) that is caused by either GD or subacute thyroiditis. We excluded patients with thyroid cancer, nodular thyroid disease, pregnancy (hyperthyroidism during pregnancy and/or within one year postpartum), drug- or iodine-induced thyroid disease, patients on oral contraceptive pills, patients who were treated before presenting to the center, and patients with incomplete data registry.

Biochemical data

The fully automated chemiluminescence immunoassay cobas e411 platform (Roche, Basel, Switzerland) was used for the measurement of serum TSH (normal range: 0.27-4.2 µIU/mL), serum fT4 (normal range: 0.93-1.7 ng/dL), T3 (normal range: 70-200 ng/dL), and TRAb (normal range: <1.75 IU/L).

Thyroid ultrasound examination

All included patients underwent US imaging of the thyroid and the neck using a linear high-frequency broadband transducer (5-18 MHz) in the radiological unit of the center using the GE Logiq E9 (GE Healthcare, Milwaukee) US machine, with a representative view on grayscale with color flow Doppler to decide on the thyroid features.

Graves' disease diagnosis

Patients with GD were included based on the presence of hyperthyroidism and one or more of the following features: detectable TRAb in the serum and evidence of ophthalmopathy and/or dermopathy [2]. By US, the findings of enlarged hypoechoic gland, thyroid echotexture exhibiting heterogeneity with increased color flow Doppler, and absence of nodularity were considered suggestive of GD [12]. Patients were categorized as GD based on the presence of TRAb with or without evidence of ophthalmopathy and/or dermopathy, and the above US features.

Subacute thyroiditis diagnosis

Patients with SAT were included based on the presence of thyrotoxicosis and clinical features of thyroidal inflammation. These were in the form of neck pain with or without jaw radiation and marked thyroid tenderness. An elevated ESR (more than 50 mL/hour), CRP (more than 3 mg/dL), and normal TRAb were documented in all patients included in the study [6,13]. The thyroid appeared focally or diffusely hypoechoic with reduced color Doppler flow on US examination [5].

**Figure 1:**
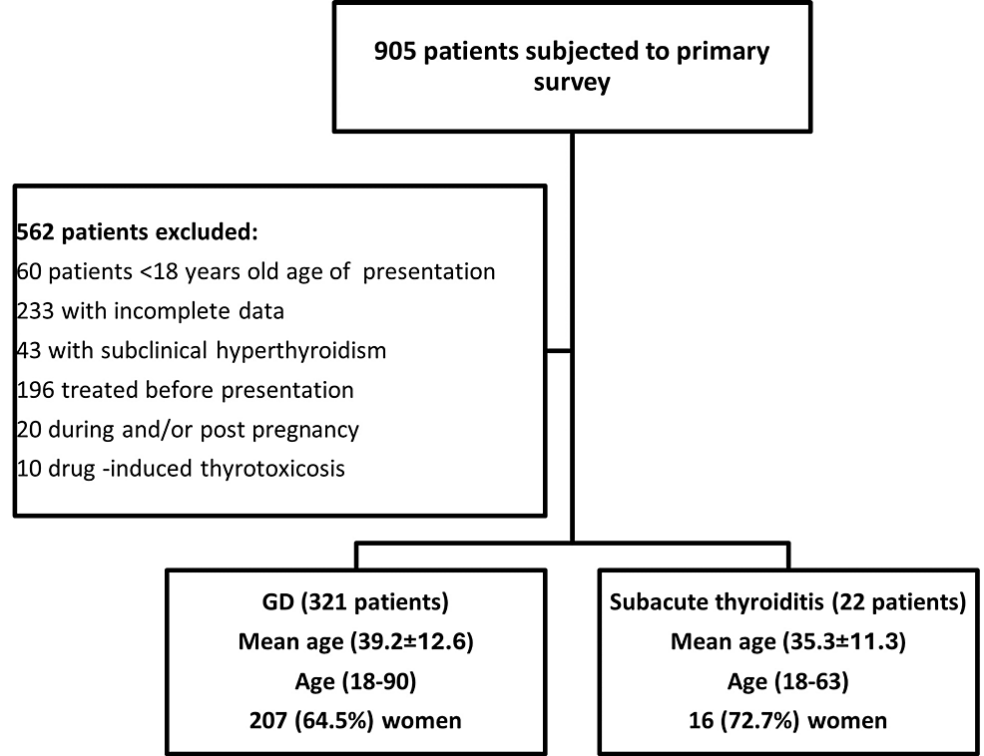
Methodology process for the patients included in the final analysis GD, Graves' disease

Figure 1 summarizes the scheme of the study. A total of 343 patients were included in the study, 321 patients with GD and 22 patients with SAT.

Statistical analysis

Data were analyzed using the Statistical Package for the Social Sciences (SPSS) version 26.0 (IBM SPSS Statistics, Armonk, NY). Categorical variables were summarized as numbers (N) and percentages (%). Continuous variables were summarized as mean±standard deviations (SDs). For comparison between GD and SAT, fT4 and T3 were used to calculate the fT4 ratio (fT4 level (ng/dL)/fT4 upper reference range (1.7 ng/dL)), T3 ratio (T3 level (ng/dL)/T3 upper reference range (200 ng/dL)), and T3/fT4 ratio (T3 level (ng/dL)/fT4 (ng/dL)). The Mann-Whitney U-test was used to determine the significance of the differences between GD and SAT (TSH, fT4, T3, TRAb, fT4 ratio, T3 ratio, and T3/fT4 ratio). Receiver operating characteristic (ROC) curves were plotted for evaluation of the accuracy of the T3/fT4 ratio in the diagnosis of GD. In the curve, the sensitivity is plotted in function of the false-positive rate (100% specificity) for different cutoff T3/fT4 ratios for GD diagnosis versus SAT. A P-value of <0.05 was defined as statistical significance for all the above comparisons.

## Results

Table 1 summarizes the baseline thyroid function data of the patients with GD and SAT. As compared to patients with subacute thyroiditis, patients with GD had significantly lower TSH and higher T3, T3 ratio, and T3/fT4 ratio (P=0.0001, 0.04, 0.04, and 0.002, respectively). No differences were found between GD and subacute thyroiditis in the fT4 and fT4 ratio.

**Table 1:** Comparison of the baseline thyroid function data between Graves' disease and subacute thyroiditis The Mann-Whitney U-test was used for comparison between the groups. *Statistically significant TSH, thyroid-stimulating hormone (µIU/mL); fT4, free thyroxine (ng/dL); T3, total triiodothyronine (ng/dL); TRAb, thyroid receptor antibodies (IU/L); fT4 ratio, fT4 level (ng/dL)/fT4 upper reference range (1.7 ng/dL); T3 ratio, T3 level (ng/dL)/T3 upper reference range (200 ng/dL); T3/fT4 ratio, T3 level (ng/dL)/fT4 (ng/dL)

Variable	Graves' disease (N=321)	Subacute thyroiditis (N=22)	P-value
TSH (µIU/mL)	0.009±0.01	0.1±0.2	*0.0001
fT4 (ng/dL)	4.1±2.0	5.1±3.7	0.6
T3 (ng/dL)	341.0±176.8	240.2±45.9	*0.04
TRAb (IU/L)	15.3±15.1	1.0±0.3	*<0.0001
fT4 ratio	2.4±1.2	3.0±2.2	0.6
T3 ratio	1.7±0.8	1.2±0.2	*0.04
T3/fT4 ratio	94.3±51.2	63.8±26.6	*0.002

**Figure 2:**
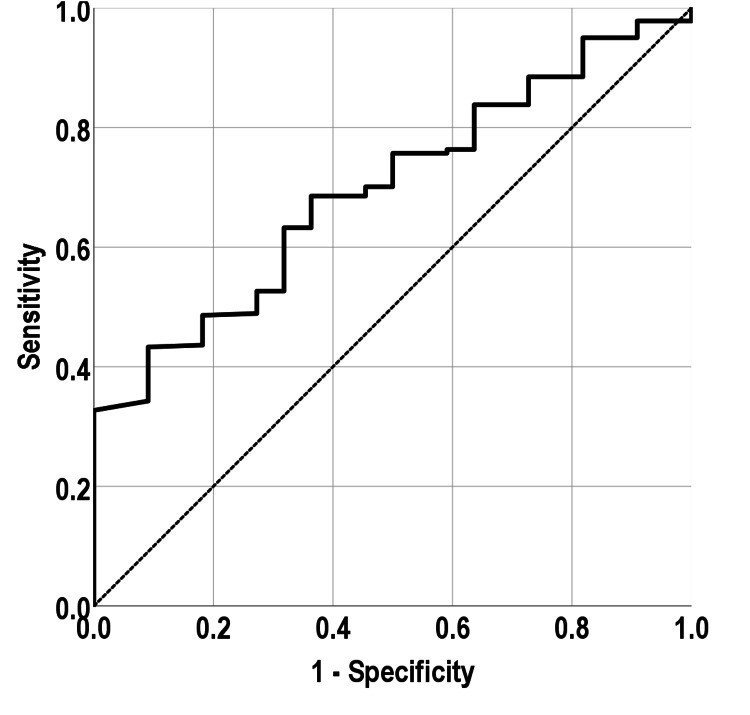
Receiver operating characteristics curve for triiodothyronine/free thyroxine ratio for Graves' disease diagnosis versus subacute thyroiditis

In this study, the T3/fT4 ratio demonstrates a ROC curve area under the curve (AUC) of 0.69, standard error of 0.04, P-value of 0.002, and 95% confidence interval of 0.6-0.7, as shown in Figure 2.

Table 2 shows the accuracy of different cutoff T3/fT4 ratios for the diagnosis of GD versus SAT. A cutoff equal to or more than 25 had 95% sensitivity and 18.1% specificity for GD with 94.4% positive predictive value. Raising the cutoff to equal or more than 100 results in a reduction of sensitivity to 32.7% but with 100% specificity and positive predictive value.

**Table 2:** Accuracy of the cutoff T3/fT4 ratio for the diagnosis of Graves' disease versus subacute thyroiditis fT4, free thyroxine; T3, total triiodothyronine

T3/fT4 ratio	Sensitivity	Specificity	Positive predictive value	Negative predictive value
≥25	95%	18.1%	94.4%	20%
≥72	70%	54.5%	95.7%	11.1%
≥100	32.7%	100%	100%	9.2%

## Discussion

This study aimed to evaluate the differences in T3/ fT4 ratios and levels among patients with GD and SAT. Our findings reveal significant variances in these parameters, especially between patients with GD and those with SAT, which underscores the potential diagnostic value of these measures in clinical practice.

The study's key findings underscore the distinct biochemical profiles observed in GD compared to SAT. Notably, patients with GD exhibited significantly lower levels of TSH and higher levels of T3, T3 ratio, and T3/fT4 ratio compared to those with subacute thyroiditis. In a study of 126 patients with thyrotoxicosis (GD, painless thyroiditis, and SAT) and 63 healthy controls, Chen et al. [14] found that untreated GD exhibited notably elevated levels of fT3 and FT4 concentrations compared to both healthy controls and individuals with painless thyroiditis. Nevertheless, there were no significant differences in fT3 and fT4 levels between untreated GD patients and those with SAT. However, the fT3/fT4 ratio was demonstrated as a useful marker for the differentiation between GD and SAT. Moreover, several studies have contributed valuable insights into examining the utility of the fT3/fT4 ratio for the differential diagnosis of GD. Izumi et al. [11] reported that despite some observed overlap, the fT3/fT4 ratio proved useful in differentiating GD and SAT. Yoshimura et al. [8] suggested that the fT3/fT4 ratio might be beneficial in distinguishing patients with GD and painless thyroiditis, especially when the fT4 value was elevated. Furthermore, Sriphrapradang et al. [15] proposed that the fT3/fT4 ratio could assist in determining the cause of thyrotoxicosis, with a higher ratio indicating GD, while a very low ratio supported the diagnosis of SAT.

Additionally, Sümbül et al. [16] revealed significant distinctions in thyroid function parameters, including TSH, fT3, and the calculated free thyroid hormone index (fTHI), between GD and SAT. The observed increase in free thyroid hormone values, particularly fTHI consistently exceeding 1 in patients with GD and remaining below 1 in patients with SAT, suggests the potential for a practical and effective method for the differential diagnosis of these two conditions. These differences underline the hyperthyroid state characteristic of GD, which is driven by TRAb stimulating the thyroid gland, as opposed to the inflammatory process seen in SAT that leads to thyroid destruction and release of thyroid hormones [17,18]. Moreover, in this study, the ROC curve analysis for the T3/fT4 ratio in GD diagnosis demonstrated an AUC of 0.69, suggesting that while the T3/fT4 ratio can help distinguish GD from SAT, it should be considered alongside other diagnostic criteria. The sensitivity and specificity of various T3/fT4 ratio cutoffs for GD diagnosis also provide valuable insights for clinical practice, indicating that a balanced approach considering both the sensitivity and specificity of these ratios together with other clinical manifestations is essential for accurate diagnosis. In a patient with thyrotoxicosis, in the absence of painful and tender thyroid, nor elevated ESR and CRP, a T3/fT4 ratio of 25 would favor GD diagnosis versus SAT. However, further research with larger, more diverse cohorts and a longitudinal approach is necessary to validate our findings and explore their implications in clinical practice more comprehensively.

The study is limited by its small sample size for SAT, which may affect the statistical power and generalizability of the findings. The exclusion of patients with toxic nodular goiter also limits the scope of the study, potentially affecting the applicability of the results to the wider spectrum of thyroid disorders. The reliance on specific cutoff values for T3/fT4 ratio diagnosis may not be universally applicable, because of the biological variability and plasma protein binding of T3.

## Conclusions

In conclusion, the T3/fT4 ratio presents as a valuable diagnostic tool in differentiating GD from SAT, with potential applications in refining the diagnostic approach to hyperthyroidism. Future studies with larger sample sizes and including a broader spectrum of thyroid disorders are warranted to further validate and refine the diagnostic cutoffs for the T3/fT4 ratio.
